# The Emergence of *bla*_NDM_-Encoding Plasmids in Enterobacteriaceae Isolated from Shared Water Resources for Livestock and Human Utilization in Central Thailand

**DOI:** 10.3390/antibiotics15010008

**Published:** 2025-12-20

**Authors:** Wipawee Songsaeng, Alongkorn Kurilung, Nuvee Prapasarakul, Thidathip Wongsurawat, Nutthee Am-In, Kittitat Lugsomya, Jenyuk Lohwacharin, Seelawut Damrongsiri, Htet Zaw Shein, Wandee Sirichokchatchawan

**Affiliations:** 1 College of Public Health Sciences, Chulalongkorn University, Bangkok 10330, Thailand; 2 Department of Biomedical Informatics, University of Arkansas for Medical Sciences, 4301 W Markham St., Little Rock, AR 72205, USA; 3 Department of Veterinary Microbiology, Faculty of Veterinary Science, Chulalongkorn University, Bangkok 10330, Thailand; 4 Center of Excellence in Diagnosis and Monitoring of Animal Pathogens (DMAP), Chulalongkorn University, Bangkok 10330, Thailand; 5 Siriraj Long-read Lab (Si-LoL), Medical Bioinformatics Lab, Siriraj Genomics, Faculty of Medicine Siriraj Hospital, Mahidol University, Bangkok 10700, Thailand; 6 Department of Obstetrics, Gynecology, and Reproduction, Faculty of Veterinary Science, Chulalongkorn University, Bangkok 10330, Thailand; 7 Faculty of Veterinary Medicine, Mahanakorn University of Technology, Bangkok 10530, Thailand; 8 Department of Environmental and Sustainable Engineering, Chulalongkorn University, Pathumwan, Bangkok 10330, Thailand; 9 Professor Aroon Sorathesn Center of Excellence in Environmental Engineering, Department of Environmental and Sustainable Engineering, Chulalongkorn University, Bangkok 10330, Thailand; 10 Sustainable Environment Research Institute, Chulalongkorn University, Bangkok 10330, Thailand

**Keywords:** carbapenemase-producing Enterobacteriaceae, multidrug resistance, plasmid conjugation, aquatic environment, Thailand

## Abstract

**Background/Objectives:** The environmental dissemination of antimicrobial-resistant Enterobacteriaceae poses a remarkable threat to public health. This study investigates the environmental presence and dissemination of carbapenemase-producing Enterobacteriaceae (CPE) in 30 important water bodies selected according to their interconnection with and utilization by livestock and community people in central Thailand. **Methods:** Water samples were collected from 30 selected water bodies. Enterobacteriaceae were isolated and screened for CPE and multidrug resistance. Carbapenemase genes (*bla*_NDM-5_, *bla*_NDM-1_ and *bla*_IMI-1_) were detected and their locations (plasmid and chromosome) determined. Plasmid types were further characterized, and conjugation experiments were performed to assess transferability among bacterial species. **Results:** From all selected samples, six isolates (20%) were identified as multidrug-resistant CPE including one *Escherichia coli*, one *Klebsiella pneumoniae* and four *Enterobacter roggenkampii* carrying *bla*_NDM-5_, *bla*_NDM-1_ and *bla*_IMI-1_ genes, respectively. The *bla*_NDM-5_ and *bla*_NDM-1_ genes were located on phage-like pO111 type plasmid and IncC plasmid, while *bla*_IMI-1_ was located on chromosomes. The plasmids also consisted of components that closely resembled those found in resistance plasmids obtained from clinical and environmental isolates worldwide. Additionally, through plasmid conjugation experiment, carbapenemase genes were transferable with a high rate among bacterial species. **Conclusions**: These findings indicated that water bodies are polluted and there is an urgent need for integrated strategies to monitor and mitigate the spread of antibiotic resistance across human, animal and environmental health domains in aquatic environments.

## 1. Introduction

The emergence of antimicrobial resistance (AMR) bacteria, especially in the environment, poses a significant threat to global public health. A specific concern is the escalating resistance against carbapenems, which are last-resort antibiotics for battling multidrug-resistant bacterial infections [1]. The World Health Organization (WHO) has classified carbapenem-resistant pathogens as a top priority for new antibiotic research, given their association with mortality rates as high as 80% [2,3]. Carbapenem resistance is primarily facilitated by the production of carbapenemase enzymes, often encoded on mobile plasmids, allowing for their rapid transmission among bacteria [1].

The carbapenemase gene, *bla*_NDM_, encodes the New Delhi metallo-β-lactamase (NDM) enzyme that confers resistance to both cephalosporins and carbapenems. The *bla*_NDM_ gene was first identified in *Klebsiella pneumoniae* isolated from a Swedish patient of Indian origin [4]. Since then, *bla*_NDM_ genes encoded in the Enterobacteriaceae, including *Escherichia coli* and *Enterobacter*, have been disseminated around the globe among human, livestock and the environment, causing a threat to public health [5,6,7,8]. In Thailand, recent studies have shown that the *bla*_NDM_ genes, for their role in carbapenem resistance, are found predominantly in clinical settings and hospital wastewater [9,10,11]. However, a reservoir in eastern Thailand has provided evidence of the environmental presence of NDM-1-producing bacteria, a finding that should sound the alarm about potential broader ecological dissemination [12]

A major concern within the spectrum of AMR is the spread of carbapenemase-producing Enterobacteriaceae (CPE) in aquatic environments. Vital for both biodiversity and human livelihood, these ecosystems are increasingly threatened by pollution from human activities, such as agriculture and urbanization. Such pollution not only harms these ecosystems but also facilitates the spread of AMR, turning water bodies into reservoirs for antibiotic-resistant pathogens [13,14,15]. The presence of CPE in water resources used for livestock, agriculture and recreational activities poses a direct and immediate threat to community health and environmental integrity. However, research on CPE in Thailand primarily targets clinical settings [10,16,17,18], with very few reports focusing on CPE in natural water resources [12,19].

In response to the evident gap in research on CPE in environmental water resources, particularly those serving multiple community uses in Thailand, this study focuses on Ratchaburi province. Given its semi-urbanization and dependence on agricultural and livestock industries, Ratchaburi’s water resources are vulnerable to AMR and CPE contamination. This research employs a targeted approach, selecting water bodies crucial for livestock, agriculture and community activities to investigate CPE presence and understand AMR dissemination mechanisms through whole-genome analysis and plasmid conjugation experiments. Adopting a One Health approach, we not only highlight the intertwined health of humans, animals and the environment but also emphasize the crucial importance of this integrated perspective. This approach is not just a strategy but a necessity in our response to the environmental challenge of carbapenem resistance in Thailand.

## 2. Results

In our study, CPE were detected in six out of thirty water resources, equating to a 20% occurrence rate. This number included the carbapenemase-producer, *bla*_IMI-1_, reported in detail in our previous study [19]. Although we have included *Enterobacter roggenkampii* in the occurrence rate, further details on these isolates were not discussed as this paper primarily focuses on the analysis of the carbapenemase gene found in plasmids.

In our initial screening we selected the isolates growth on CHROMagar, and 48 isolates were identified as Enterobacteriaceae. After applying the Carba NP test, 19 isolates exhibited positive results. Among these positive isolates, we found that only a fraction exhibited strong resistance to carbapenems. Antibiotic susceptibility testing indicated only six isolates considered to be carbapenemase-producers exhibited multidrug resistance, including resistance to carbapenems (MIC value ranging from ≥4 µg/mL to >16 µg/mL) and β-lactam antibiotics like third- and fourth-generation cephalosporins. In addition to carbapenem and β-lactam antibiotics resistance, *K. pneumoniae* showed resistance to colistin, underscoring the potential to undermine the efficacy of last-resort antibiotics in Table 1.

Further investigation through polymerase chain reaction (PCR) and whole-genome sequencing (WGS) identified the carbapenemase genes *bla*_NDM-5_ and *bla*_NDM-1_ within the plasmids along with other antibiotic-resistance genes of *E. coli* and *K. pneumoniae* isolates, respectively (Table 2). Interestingly, although both isolates carried carbapenemase genes, a few β-lactam agents showed intermediate susceptibility in the phenotypic testing. This phenotype has been reported previously and may reflect biological variation in gene expression and resistance mechanisms.

**Figure 1:**
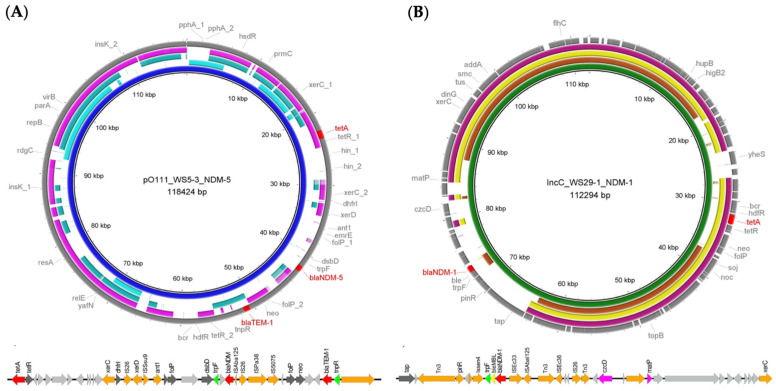
Comparative circular maps of *bla*_NDM-5_ and *bla*_NDM-1_-carrying plasmids generated using BRIG. (**A**) The rings, from inner to outer, present the plasmids pRWS531 from *E. coli* from this study (GenBank acc. no. CP110512), pO111_2 DNA (GenBank acc. no. AP010962) from *E. coli* strain11128, pMTY9754_pO111 (GenBank acc. no. AP026489) from *E. coli* isolate and p16EC-pO111 (GenBank acc. no. MN086777) from *E. coli* isolate. The outer circle with gray and red arrows denotes the annotation of plasmid pRWS531. (**B**) The rings, from inner to outer, represent the plasmids pRWS291 from *K. pneumoniae* from this study (GenBank acc. no. CP110518), pNDM-KN (GenBank acc. no. JN157804) from *K. pneumoniae* isolate, IncA/C-LS6 (GenBank acc. no. JX442976) and pET8.1-IncAC2 (GenBank acc. no. CP043215) from *Salmonella enterica* isolate. The outer circle with gray and red arrows denotes the annotation of plasmid pRWS291. Below both figures, (**A**,**B**), are the genetic environments of the *bla*_NDM-5_-carrying plasmid of *E. coli* and the *bla*_NDM-1_-carrying plasmid of *K. pneumoniae* and the light gray arrows represent the hypothetical proteins.

Comparative genomics have linked a clinical NDM-5-producing *E. coli* strain from China with a non-clinical *E. coli* strain found in our study, stressing the global challenge of AMR spread (Figure 1). Similarly, a *K. pneumoniae* isolate, identified as ST6316 and closely related to the ST1310 clinical strain, indicates possible transmission from clinical settings to natural water bodies, raising concerns over the resistance of these bacteria to crucial antibiotics, shown in Table 2 and Supplementary Figure S1 [20,21,22]. The multi-locus sequence type (MLST) analysis further supports the epidemiological connection between environmental isolates in our study and those associated with severe clinical outcomes, suggesting the movement of highly resistant bacteria from healthcare environments to natural settings [23,24].

In addition, our plasmid analysis revealed that *E. coli* and *K. pneumoniae* isolates, harboring carbapenemase genes on their plasmids, were among the contaminants identified in selected natural water resources. Specifically, the *bla*_NDM-5_ gene was found on a plasmid with an uncommon replicon pO111, which is also known as phage-like pO111 plasmid. The comparative analysis highlighted that this plasmid shares high nucleotide sequence similarity with pO111-2DNA from clinical isolates in Osaka (GenBank accession no. AP010962) [25] and with plasmids from food-producing animals in Japan and China (GenBank accession no. AP026489 and MN086777) (Figure 1A), despite these not carrying *bla*_NDM_ genes [25,26,27].

In addition, the *K. pneumoniae* in our study was found to carry the *bla*_NDM-1_ gene on a 112 kb IncC plasmid (Figure 1B), previously linked to multi-antibiotic resistance, including carbapenems, aminoglycosides, and fluoroquinolones. This plasmid shares identical regions with the pNDM-KN plasmid from a Kenyan hospital (GenBank accession no. JN157804) and shows great similarity to plasmids initially found in clinical isolates and food-producing animals across different regions [28,29,30,31], suggesting a concerning potential for broad host-range plasmid-mediated antibiotic resistance spread.

The genetic map of the phage-like pO111 plasmid, featuring IS*Aba*125 sequences and N-anthranilate isomerase (trpF), further highlights a unique genetic environment (Figure 1A). While the genetic structure of the IncC plasmid includes small mobile genetic elements, with the bleomycin resistance protein (*ble*_MBL_) and trpF identified upstream of *bla*_NDM-1_, it aligns with genetic backbones observed in clinical IncC plasmids (Figure 1B). The region of the genomic environment carrying *trpF*, *ble*_MBL_ and *bla*_NDM-1_ on the IncC plasmid in this study was found identical to the typical region observed in IncC plasmid backbones carrying *bla*_NDM-1_ in clinical isolates from other studies [28,32].

In our study, conjugation experiments conducted with *E. coli* J53 revealed that both *E. coli* and *K. pneumoniae* isolates were able to transfer carbapenem resistance genes effectively. The transconjugants for both *E. coli* and *K. pneumoniae* were able to grow normally on Luria–Bertani (LB) plates containing meropenem (2 μg/mL) and sodium azide (100 μg/mL). The transfer frequency rate was approximately 10^−3^ to 10^−4^ for both strains, with *K. pneumoniae* isolate exhibiting higher frequency at 5.52 × 10^−3^, followed by *E. coli* isolate with the frequency of 2.24 × 10^−4^ Table 2.

## 3. Discussion

In this study, CPE were detected in 20% of the sampled water bodies, a proportion higher than that reported in comparable studies conducted in natural river systems in Switzerland, where CPE occurrence was approximately 10% [33]. This elevated detection rate suggests that shared water resources in central Thailand may represent an important and underrecognized environmental reservoir for carbapenem-resistant bacteria. The relatively high incidence observed in this study likely reflects local anthropogenic pressures, including agricultural runoff as well as livestock and municipal waste inputs, which are known drivers of antimicrobial resistance dissemination in aquatic environments [34].

All CPE isolates identified in this study exhibited multidrug resistance (MDR), underscoring the clinical and public health relevance of environmental CPE. Enterobacteriaceae commonly acquire carbapenem resistance through the production of carbapenemase enzymes capable of degrading β-lactam antibiotics, a mechanism that frequently co-occurs with resistance to multiple antimicrobial classes [35]. Consistent with previous reports, clinical CPE isolates typically demonstrate broader resistance profiles compared with non-CPE strains [36], raising concerns that environmental reservoirs may contribute to the persistence and spread of difficult-to-treat infections beyond healthcare settings.

Importantly, the presence of a carbapenemase gene does not necessarily result in uniform or high-level phenotypic resistance. Factors such as promoter strength, regulatory elements, gene copy number and environmental conditions can substantially influence gene expression and enzymatic activity [37]. In addition, high-level resistance to carbapenems and other β-lactam antibiotics often requires complementary mechanisms, including porin loss or efflux pump overexpression. In the absence of these additional mechanisms, β-lactams may still reach penicillin-binding proteins, resulting in intermediate susceptibility despite the presence of carbapenemase genes [38]. These observations help explain the phenotypic variability observed among carbapenemase-producing isolates recovered from environmental sources.

Beyond phenotypic resistance, the localization of carbapenemase genes on plasmids represents a critical concern for antimicrobial resistance dissemination. Our findings demonstrate that *bla*_NDM_ genes were carried on highly mobile plasmids, highlighting the significant role of horizontal gene transfer in spreading resistance determinants across bacterial populations [39]. Although high-mobility plasmids are not traditionally associated with *bla*_NDM_ genes, they have been shown to harbor a wide range of resistance elements. The ability of *bla*_NDM-1_ to disseminate via IncC plasmids across diverse genetic backgrounds represents a substantial global health threat. Similarly, the detection of *bla*_NDM-5_ on a phage-like pO111 plasmid, a rare occurrence, highlights the potential for the extensive and rapid dissemination of this resistance gene and emphasizes the unique role of unconventional plasmid backbones in antimicrobial resistance evolution.

The genetic organization of resistance loci within these plasmids further supports their mobilization potential. In this study, the genetic maps indicated that *bla*_NDM_ genes were embedded within mobilizable plasmid backbones, which may enhance their transmission among environmental bacterial strains and across ecological niches. From a One Health perspective, the presence of such highly transferable resistance elements in water bodies shared by humans, livestock, and agricultural activities increases the likelihood of cross-sector transmission and complicates efforts to contain antimicrobial resistance.

Finally, the conjugation experiments performed in this study demonstrated a high capacity for horizontal gene transfer between *E. coli* and *K. pneumoniae* isolates [40]. Subsequent analyses confirmed that transconjugants successfully acquired NDM resistance genes, providing direct evidence that NDM-encoding plasmids can move between bacterial hosts and propagate carbapenem resistance within environmental microbial communities [41]. Environmental factors such as nutrient availability, microbial density and pollutant exposure may further accelerate the spread of *bla*_NDM_ genes, increasing the risk of the widespread dissemination of multidrug-resistant bacteria in aquatic ecosystems [34]. Together, these findings highlight the need for integrated environmental surveillance strategies that incorporate plasmid-level monitoring to better anticipate and mitigate the spread of carbapenem resistance across human, animal and environmental health domains.

## 4. Materials and Methods

### 4.1. Sampling and Detection of CPE

**Figure 2:**
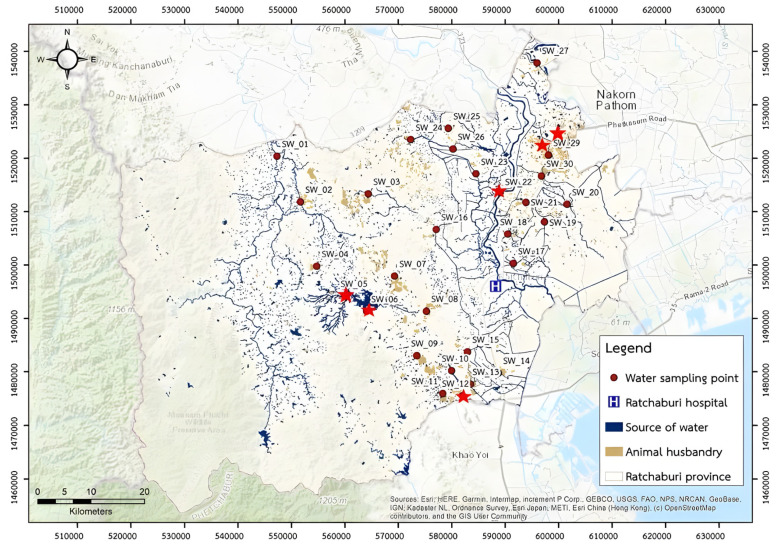
Map of Ratchaburi province showing bodies of water, the sample locations and carbapenemase genes’ detection status. The red dots indicated the sample locations, and red stars indicated the locations where CPE were detected. These sampling sites were the same sites with our previous study [19].

Thirty water resources across Ratchaburi province were selected and the samples were collected from different sampling sites according to our previous study [19]. These sampling sites were chosen due to their intensive use in livestock farming, agriculture and community activities, representing a cross-section of the province’s water resources (Figure 2). From each location, one liter of water was collected in sterile conditions, immediately cooled on ice, and processed in the laboratory within 24 h. The water samples were filtered through sterile 0.45 μm membrane filters. The filters were incubated at 37 °C for 24 h in 10 mL of EE Broth Mossel (Himedia, Thane, India) for enrichment purposes. One loopful of each enrichment culture was inoculated on CHROMagar™ mSuperCARBA (CHROMagar™, Paris, France) to isolate carbapenemase producers, and incubated for 24 h at 37 °C [42]. All colonies with different morphologies and colors were selected for bacteria species’ identification using matrix-assisted laser desorption ionization–time of flight mass spectrometry (MALDI-TOF MS).

### 4.2. Antimicrobial Susceptibility

The Enterobacteriaceae isolates were confirmed for carbapenemase production using the Carba NP test (Biomerieux, Marcy-l’Étoile, France). The antibiotic susceptibility test (AST) was performed using the broth microdilution method (sensitizer plates by Thermo Fisher Scientific, Waltham, MA, USA), and the minimal inhibitory concentration (MIC) was interpreted according to the Clinical and Laboratory Standards Institute guideline [43], with *E. coli* ATCC 25922 as the quality control strain. The guideline indicated that the value of ≥4 µg/mL of meropenem, imipenem and doripenem, and the value of ≥2 µg/mL of ertapenem are considered to be resistant. In addition, for all isolates, PCR was performed to detect carbapenemase genes such as *bla*_IMP_, *bla*_VIM_, *bla*_SPM_, *bla*_KPC_, *bla*_OXA-48_, *bla*_NDM_, *bla*_AIM_, *bla*_BIG_, *bla*_SIM_ and *bla*_DIM_. The primer sequence and PCR conditions were previously described by [44] and outlined in Supplementary Table S1.

### 4.3. WGS and Plasmid Conjugation Analysis

The DNA extraction for positive isolates with carbapenemase genes was performed and the genomic sequences were carried out using both the Illumina NovaSeq 6000 platform (Illumina, San Diego, CA, USA) and long-read MinION on a R9.4 Spot On flow cell (GridION, Oxford Nanopore Technologies, UK). The details of genome analysis for antibiotic resistance genes, MLST and genetic context of *bla*_NDM_ are included in Supplementary Materials.

WGS identified the plasmid incompatibility groups, and the conjugation experiments were conducted with the broth mating method using CPE isolates as donors and sodium azide-resistant *E. coli* J53 as the recipient. Donor strains and recipients *E. coli* J53 were mixed at a ratio of 1:1 in a LB broth and incubated overnight at 35 °C. Transconjugants were selected on Luria–Bertani agar plates (HiMedia Laboratories, Maharashtra, India) containing meropenem (2 μg/mL) and sodium azide (100 μg/mL). The species identification of transconjugants was performed using MALDI-TOF MS. The confirmation of transfer was determined by PCR to detect the carbapenemase genes among the transconjugants [40,45].

## 5. Conclusions

Our research is among the first environmental report in Thailand to highlight the critical issue of contamination in water bodies located near hospitals, livestock farms and residential areas. We found carbapenemase genes in environmental isolates that closely resemble those identified in clinical settings, demonstrating their persistence and widespread dissemination locally and globally. Although determining the exact origins of these genes remains challenging, our findings support previous evidence that human activities, livestock production and recreational water use contribute to the accumulation and spread of antimicrobial resistance in aquatic ecosystems. These results raise concerns about community exposure to resistant bacteria and the potential for cross-sector AMR transmission. Our findings emphasize the urgent need for integrated surveillance and intervention strategies aligned with the One Health approach to mitigate the environmental spread of these pathogens. In this study, the antimicrobial susceptibility testing focused on a targeted panel of antibiotics provided in the automated MIC system. This panel includes the most clinically relevant β-lactams and carbapenems used across human and veterinary medicine, aligning with the study’s objective to detect carbapenemase-producing isolates. Nevertheless, expanding the antibiotic panel to include a broader range of agents available on the market would offer a more comprehensive resistance profile and should be considered in future work. Finally, the study’s scope, limited to a single-time sample collection from one Thai region, suggests that the findings may not fully represent the national scenario, indicating the necessity for more extensive, countrywide research on CPE’s environmental spread.

## Figures and Tables

**Table 1:** Species, sources and antimicrobial resistance profiles of carbapenemase-producing isolates from water bodies surrounding the farms and communities in central Thailand.

Isolate No.	Bacteria Species	Location of Water Sources	Carbapenems				Other Antimicrobials															
			DOR	IPM	MEM	ETP	AMK	AMC	AMP	FEP	FOX	CAZ	CTX	SAM	TZP	CST	CIP	CRO	LVX	GEN	NET	SXT
WS_5-3	*Escherichia coli*	Next to pig farms	R	I	R	R	S	R	I	R	I	R	R	R	I	S	I	R	R	I	S	R
WS_29-1	*Klebsiella pneumoniae*	Within community	R	I	R	R	S	R	I	R	S	R	R	R	I	R	S	R	S	I	S	R

Abbreviations: DOR—doripenem, IPM—imipenem, MEM—meropenem, ETP—ertapenem, AMK—amikacin, AMC—amoxicillin-clavulanic acid, AMP—ampicillin, FEP—cefepime, FOX—cefoxitin, CAZ—ceftazidime, CTX—cefotaxime, SAM—ampicillin-sulbactam, TZP—piperacillin-tazobactam, CST—colistin, CIP—ciprofloxacin, CRO—ceftriaxone, LVX—levofloxacin, GEN—gentamicin, NET—netilmicin, SXT—trimethoprim-sulfamethoxazole, R—resistance, I—intermediate, and S—susceptible. The details of resistance pattern and genetic characteristics of *E. roggenkampii* isolates were presented in our previous report [19].

**Table 2:** Summary of features associated with carbapenemase-producing *E. coli and K. pneumoniae* strains cultured from shared water bodies surrounded farms and communities in Ratchaburi province, Thailand.

Isolate	Bacterial Species	Carbapenemase Genes	Other AMR Genes ^a^	Gene Location (Replicon Type)	Transfer Ability Rate	ST	GenBank Accession *
WS5-3	*Escherichia coli*	*bla*_NDM-5_	*aph(6)-Id*, *aadA2*, *aph(3″)-Ib*, *dfrA14*, *sul2*, *sul1*, *dfrA12*, *qnrS1*, *tet(A)*, *bla*_TEM-1_	Plasmid (pO111)	2.24 × 10^−4^	ST 4538	CP110512
WS29-1	*Klebsiella* *pneumoniae*	*bla*_NDM-1_	*aph (3″)-lb*, *mph(A)*, *aph(6)-ld*, *sul2*, *tet(A)*, *bla*_SHV_	Plasmid (IncC)	5.52 × 10^−3^	ST 6316 ^b^	CP110518

^a^ Antibiotic resistance genes, *aph* Aminoglycoside phosphotransferase, *aad* Aminoglycoside adenylyl transferase, *dfrA* Dihydrofolate, *sul* Sulfonamide resistance gene, *qnrS* Quinolone resistance, *tet(A)* Tetracycline resistance, *bla* beta-lactamases, *mph* Macrolide phosphotransferases, Fosfomycin resistance, and ^b^ new sequence type. * The GenBank accession numbers are specified for plasmids carrying *bla*_NDM-5_ and *bla*_NDM-1_ in this study only.

## Data Availability

The datasets generated and analyzed during the current study are available in the National Library of Medicine (NCBI database) under the BioProjects: PRJNA895848 and PRJNA895725.

## Supplementary Materials

The following supporting information can be downloaded at: https://www.mdpi.com/article/10.3390/antibiotics15010008/s1. Table S1: Primers for carbapenem-resistant genes; Figure S1: Population snapshot of *Klebsiella pneumoniae*. Refs. [1,33,44,46,47,48] are cited in Supplementary Materials.

## Abbreviations

The following abbreviations are used in this manuscript:

AMR	Antimicrobial Resistance
AST	Antibiotic Susceptibility Test
*bla*_NDM_	New Delhi Metallo-β-Lactamase Gene
CPE	Carbapenemase-Producing Enterobacteriaceae
LB	Luria–Bertani
MALDI-TOF MS	Matrix-Assisted Laser Desorption Ionization-Time of Flight Mass Spectrometry
MDR	Multidrug Resistance
MIC	Minimal Inhibitory Concentration
MLST	Multi-Locus Sequence Type
NDM	New Delhi Metallo-β-lactamase
PCR	Polymerase Chain Reaction
WGS	Whole-Genome Sequencing
WHO	World Health Organization
