# Supplementary material for: The Emergence of blaNDM-Encoding Plasmids in Enterobacteriaceae Isolated from Shared Water Resources for Livestock and Human Utilization in Central Thailand
Source: Antibiotics (Basel). 2025 Dec 20;15(1):8. doi: 10.3390/antibiotics15010008 (PMC12837215; doi:10.3390/antibiotics15010008)
Supplement: Supplementary file 1 [file antibiotics-15-00008-s001.zip › antibiotics-3985305-supplementary.pdf]

Supplementary Materials S1

Supplementary method

The DNA extraction for positive isolates with carbapenemase genes was performed using a ZymoBIOMICS DNA extraction kit (CA, United States). DNA quality was evaluated using NanoDrop (A260/A280 and A260/A230 ratios for purity) and Qubit (for right concentration) before sequencing. The genomic sequences were carried out using both the Illumina NovaSeq 6000 platform and long-read MinION on a R9.4 Spot On flow cell (Oxford Nanopore Technologies). Adapter trimming software (Trimmomatic) was used for quality control for reading the trimming and the de novo hybrid assembly of short reads and long reads was performed using Unicycler (v0.4.8). The whole genome sequence was annotated using the prokaryotic gene prediction tool Prokka 1.14.6 with standard settings [1]. The analysis pipeline at the Center for Genomic Epidemiology (www.genomicepidemiology.org) was employed to analyze the antibiotic resistance genes and plasmid incompatibility groups using ResFinder and PlasmidFinder [2].

Multilocus sequence typing (MLST) was performed using the MLST database (Achtman's and Institut Pasteur's schemes). Plasmid comparisons and alignments were performed by using the Basic Local Alignment Search Tool (BLAST) at the National Center for Biotechnology Information (NCBI) (https://blast.ncbi.nlm.nih.gov/Blast.cgi) [3]. The annotated plasmids carrying carbapenemase genes were compared with reference plasmids belonging to the same incompatibility groups using the BLAST ring image generator (BRIG). The genetic environment of all resistance genes was investigated using Artemis and Easyfig [4,5].

Table S1. Primers for carbapenem-resistant genes (Poirt et al., 2011 [6])

Template	Amplicon size (bp)	Sequence (5′-3′)	Reference
***bla*KPC**	798	5′-CGTCTAGTTCTGCTGTCTTG -3′<br>5′CTTGTCATCCTTGTTAGGCG -3′	(Poirel, L. et. al., 2011) [6]
***bla*IMP**	232	5′-GGAATAGAGTGGCTTAAYTCTC -3′<br>5′-GGTTTAAYAAAACAACCACC -3′	(Poirel, L. et. al., 2011) [6]
***bla*VIM**	390	5′-GATGGTGTTTGGTCGCATA -3′<br>5′-CGAATGCGCAGCACCAG -3′	(Poirel, L. et. al., 2011) [6]
***bla*NDM**	621	5′-GGTTTGGCGATCTGGTTTTC -3′<br>5′-CGGAATGGCTCATCACGATC -3′	(Poirel, L. et. al., 2011) [6]
***bla*Oxa-48**	438	5′-GCGTGGTTAAGGATGAACAC -3′<br>5′-CATCAAGTTCAACCCAACCG -3′	(Poirel, L. et. al., 2011) [6]

***bla*SPM**	271	5′-AAAATCTGGGTACGCAAACG -3′<br>5′-ACATTATCCGCTGGAACAGG -3′	(Poirel, L. et. al., 2011) [6]
***bla*AIM**	322	5′-CTGAAGGTGTACGGAAACAC -3′<br>5′-GTTCGGCCACCTCGAATTG -3′	(Poirel, L. et. al., 2011) [6]
***bla*BIG**	537	5′-TATGCAGCTCCTTTAAGGGC -3′<br>5′-ATCATTGGCGGTGCCGTACAC -3′	(Poirel, L. et. al., 2011) [6]
***bla*GIM**	477	5′-TCGACACACCTTGGTCTGAA -3′<br>5′-AACTCCAACTTTGCCATGC -3′	(Poirel, L. et. al., 2011) [6]
***bla*SIM**	570	5′-TACAAGGGATTCGGCATCG -3′<br>5′-TAATGGCCTGTTCCCATGTG -3′	(Poirel, L. et. al., 2011) [6]
***bla*DIM**	699	5′-GCTTGTCTTCGCTTGCTAACG-3′<br>5′-CGTTCGGCTGGATTGATTTG-3′	(Poirel, L. et. al., 2011) [6]

Supplementary Materials S2

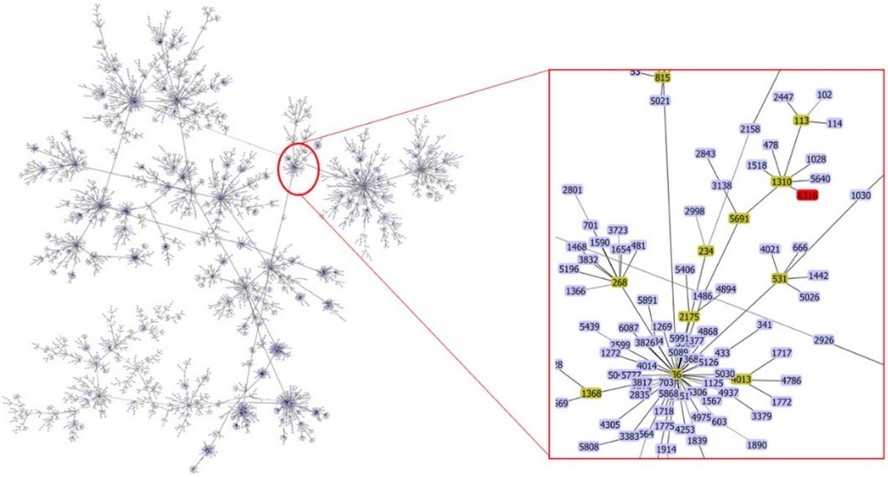

Figure S1 Population snapshot of *Klebsiella pneumoniae*. Each number indicates a sequence type (ST); the yellow highlight represents group founder, and the light blue highlight represents the common node. The newly detected sequence type, ST6316 (highlighted in red) from this study, is linked with the existing ST1310.

1. Huang, J.; Zhu, J.; Gong, D.; Wu, L.; Zhu, Y.; Hu, L. Whole genome sequence of EC16, a blaNDM-5-, blaCTX-M-55-, and fosA3-coproducing Escherichia coli ST167 clinical isolate from China. *Journal of Global Antimicrobial Resistance* **2022**, *29*, 296-298, doi:https://doi.org/10.1016/j.jgar.2022.04.001.

2. Bleichenbacher, S.; Stevens, M.; Zurfluh, K.; Perreten, V.; Endimiani, A.; Stephan, R.; Nüesch-Inderbinen, M. Environmental dissemination of carbapenemase-producing Enterobacteriaceae in rivers in Switzerland. *Environmental Pollution* **2020**, *265*, 115081, doi:https://doi.org/10.1016/j.envpol.2020.115081.
3. Zhao, Q.; Berglund, B.; Zou, H.; Zhou, Z.; Xia, H.; Zhao, L.; Nilsson, L.E.; Li, X. Dissemination of bla(NDM-5) via IncX3 plasmids in carbapenem-resistant Enterobacteriaceae among humans and in the environment in an intensive vegetable cultivation area in eastern China. *Environ Pollut* **2021**, *273*, 116370, doi:10.1016/j.envpol.2020.116370.
4. Alikhan, N.; Petty, N.; Zakour, N.; Beatson, S. BLAST Ring Image Generator (BRIG): simple prokaryote genome comparisons. *BMC Genomics* **2011**, *12*.
5. Sullivan, M.; Petty, N.; Scott, B. Easyfig: a genome comparison visualiser. *Bioinformatics* **2011**, *27*, 2.
6. Poirel, L.; Walsh, T.R.; Cuvillier, V.; Nordmann, P. Multiplex PCR for detection of acquired carbapenemase genes. *Diagn. Microbiol. Infect. Dis.* **2011**, *70*, 119–123. https://doi.org/10.1016/j.diagmicrobio.2010.12.002. PMID: 21398074.
